# Ocular Comorbidities Contributing to Death in the US

**DOI:** 10.1001/jamanetworkopen.2023.31018

**Published:** 2023-08-25

**Authors:** Ryan S. Huang, Andrew Mihalache, Marko M. Popovic, Peter J. Kertes, David T. Wong, Rajeev H. Muni

**Affiliations:** 1 Temerty Faculty of Medicine, University of Toronto, Toronto, Ontario, Canada; 2 Department of Ophthalmology and Vision Sciences, University of Toronto, Toronto, Ontario, Canada; 3 John and Liz Tory Eye Centre, Sunnybrook Health Sciences Centre, Toronto, Ontario, Canada; 4 Department of Ophthalmology, St Michael’s Hospital, Unity Health Toronto, Toronto, Ontario, Canada

## Abstract

This cross-sectional study estimates the prevalence of ocular comorbidities contributing to death in the US, stratified by age, sex, and race and ethnicity.

## Introduction

Globally, it is estimated that more than 200 million individuals have visual impairment.^1^ Indeed, poorer visual functioning has been associated with higher mortality rates.^2,3^ Currently, data are lacking on ocular comorbidities as contributing causes of US deaths. To our knowledge, this population-based study is the largest to provide comprehensive estimates of ocular comorbidities contributing to death in the US.

## Methods

This cross-sectional study was conducted using mortality records from the US Centers for Disease Control and Prevention (CDC). Mortality data were based on death certificates validated by medical examiners. Race and ethnicity was identified based on information from an informant or observation; these data were included to analyze differences in ocular comorbidities contributing to death across various racial and ethnic groups. All US deaths that occurred between January 2000 and December 2019 and listed ocular disease as a contributor were extracted. *International Statistical Classification of Diseases, Tenth Revision* (*ICD-10*) codes H00 to H59 encompassing diseases of the eye and ocular adnexa were used to select patients. Because publicly available data were used, the University of Toronto deemed this study exempt from institutional ethics approval and waived informed consent. We followed the STROBE reporting guideline.

The number of ocular comorbidities contributing to US deaths was computed per 100 000 deaths annually (2000-2019). Strata-specific frequencies were calculated by age, sex, and race and ethnicity. Univariable linear regression models were used to examine trends in ocular comorbidity prevalence over the study period. *P* < .05 (2-tailed) was considered statistically significant. Statistical analyses were conducted on April 26, 2023, using SAS, version 9.4 (SAS Institute).

## Results

Between 2000 and 2019, 51 125 902 deaths were reported in the US, with higher rates among men (50.1%), White individuals (79.6%), and those aged 80 to 89 years (28.5%) (Table 1). There were 51 256 deaths (1 per 1000) associated with a medically confirmed ocular comorbidity. These deaths were observed most often among individuals aged 90 to 99 years (35.3%) and women (62.2%). Most ocular comorbidities contributing to death were observed among White individuals (81.8%) compared with other racial and ethnic groups (American Indian or Alaska Native [0.5%], Asian or Pacific Islander [1.7%], Black [11.2%], or Hispanic [4.8%]). The ocular comorbidity most frequently contributing to death was binocular blindness in American Indian or Alaska Native and Hispanic individuals.

**Table 1.** Prevalence of Ocular Comorbidities Contributing to Death in the US Population From 2000 to 2019, Stratified by Age, Sex, and Race and Ethnicity

Characteristic	Total deaths in the US general population, No. (%)	Ocular comorbidities contributing to death			
		No. (%)	Per 100 000 total deaths, No. (95% CI)	Most frequent
Sex				
Male	25 604 828 (50.1)	19 400 (37.8)	75.8 (73.3-78.3)	Glaucoma
Female	25 521 074 (49.9)	31 856 (62.2)	124.8 (119.4-130.8)	Glaucoma
Race and ethnicity				
American Indian or Alaska Native	304 548 (0.6)	251 (0.5)	82.4 (72.9-91.5)	Binocular blindness
Asian or Pacific Islander	1 062 731 (2.1)	879 (1.7)	82.7 (77.9-90.6)	Glaucoma
Black	6 031 916 (11.8)	5752 (11.2)	95.4 (88.8-101.8)	Glaucoma
Hispanic	3 055 686 (6)	2457 (4.8)	80.4 (74.2-83.5)	Binocular blindness
White	40 671 021 (79.6)	41 917 (81.8)	103.1 (99.3-107.2)	Macular degeneration
Age, y				
<50	5 328 827 (10.4)	2092 (4.1)	39.0 (37.3-40.7)	Binocular blindness
50-59	4 888 186 (9.6)	1764 (3.4)	36.1 (34.4-37.9)	Binocular blindness
60-69	7 473 779 (14.6)	3296 (6.4)	44.1 (41.8-46.2)	Binocular blindness
70-79	10 907 392 (21.3)	6705 (13.1)	61.5 (58.1-64.3)	Glaucoma
80-89	14 553 919 (28.5)	17 306 (33.8)	118.9 (111.7-126.4)	Glaucoma
90-99	7 508 189 (14.7)	18 093 (35.3)	241.0 (230.5-258.7)	Cataract
>99	465 610 (0.9)	2000 (3.9)	429.5 (406.7-456.4)	Cataract
Total	51 125 902 (100)	51 256 (100)	100.3 (94.5-104.5)	Glaucoma

From 2000 to 2019, the number of ocular comorbidities contributing to death decreased overall by 1.7% (2849 to 2801; Table 2). Among Black and White individuals, decreases of 17.1% (369 to 306) and 6.5% (2366 to 2213) were observed, respectively. A significant trend in increased prevalence of ocular comorbidities contributing to death was observed among American Indian and Alaska Native and Hispanic individuals, with increases of 166.7% (6 to 16) and 194.0% (67 to 197), respectively (*P* < .001).

**Table 2.** Change in the Number of Ocular Comorbidities Contributing to Death in the US Population From 2000 to 2019

Characteristic	Ocular comorbidities contributing to death		*P* value^a^
	% Change from 2000 to 2019	Annual change per 100 000 total deaths	
Sex			
Male	12.2	−1.8 (−2.3 to −1.3)	<.001
Female	−9.3	−0.4 (−0.8 to 0)	.08
Race and ethnicity			
American Indian or Alaska Native	166.7	1.6 (0.5 to 2.7)	<.001
Asian or Pacific Islander	68.3	−1.2 (−2.0 to −0.3)	<.001
Black	−17.1	−2.0 (−2.7 to −1.2)	<.001
Hispanic	194.0	1.3 (0.8 to 1.8)	<.001
White	−6.5	−1.1 (−1.5 to −0.7)	<.001
Age, y			
<50	−6.0	−0.1 (−0.4 to 0.2)	.47
50-59	40.0	0 (−0.3 to 0.3)	.83
60-69	58.4	0 (−0.4 to 0.4)	.91
70-79	−14.9	−0.7 (−1.1 to −0.2)	.005
80-89	−26.8	−2.4 (−3.0 to −1.9)	<.001
90-99	15.4	−4.5 (−5.7 to −3.2)	<.001
>99	39.0	−3.3 (−7.4 to 0.8)	.11
Total	−1.7	−1.2 (−1.6 to −0.7)	<.001

^a^ *P* values for trends in ocular comorbidities contributing to death over time were computed using univariable linear regression models.

## Discussion

We observed substantial racial and ethnic disparities in the prevalence of ocular comorbidities contributing to US deaths from 2000 to 2019, with notable increases among American Indian or Alaska Native and Hispanic individuals. A previous study reported that 63% of Latino individuals with eye disease had no reported history of disease^4^; this group has the highest rates of uninsured individuals in the US.^5^ Given the decrease in uninsured individuals,^4^ it is plausible that improved disease documentation over the last 2 decades partly accounted for the increase in ocular comorbidities contributing to death.

This cross-sectional study has some limitations. A previous study validated death certificate reporting in the US, with excellent results for Black and White populations but worse results for American Indian or Alaska Native and Asian individuals.^6^ However, adjustment for misclassifications did not notably affect mortality differentials.^6^ To mitigate misclassification risks in this analysis, we analyzed data from 2000 to 2019, representing the longest contiguous period for which the CDC utilized the same *ICD-10* codes. Furthermore, the large sample size and population-based nature of this study allowed for generalizability of our findings to the US civilian population.

## Notes

Supplement.
Data Sharing Statement
