# Supplementary material for: Ocular Comorbidities Contributing to Death in the US
Source: JAMA Netw Open. 2023 Aug 25;6(8):e2331018. doi: 10.1001/jamanetworkopen.2023.31018 (PMC10457708; doi:10.1001/jamanetworkopen.2023.31018)
Supplement: Supplement. — Data Sharing Statement [file jamanetwopen-e2331018-s001.pdf]

# Data Sharing Statement

Huang. Ocular Comorbidities Contributing to Death in the US. *JAMA Netw Open*. Published August 25, 2023. doi:10.1001/jamanetworkopen.2023.31018

**Data**
**Data available:** Yes
**Data types:** Data (not involving human participants)
**How to access data:** https://www.cdc.gov/nchs/data_access/vitalstatsonline.htm#Mortality_Multiple
**When available:** With publication

**Supporting Documents**
**Document types:** None

**Additional Information**
**Who can access the data:** Anyone requesting data
**Types of analyses:** Any purpose
**Mechanisms of data availability:** Publicly Available
